# Supplementary material for: Broadband multi-magnon relaxometry using a quantum spin sensor for high frequency ferromagnetic dynamics sensing
Source: Nat Commun. 2020 Oct 16;11:5229. doi: 10.1038/s41467-020-19121-0 (PMC7568545; doi:10.1038/s41467-020-19121-0)
Supplement: Supplementary file 1 — Supplementary Information [file 41467_2020_19121_MOESM1_ESM.pdf]

Broadband multi-magnon relaxometry using a quantum spin sensor for high frequency ferromagnetic dynamics sensing: Supplementary Information

Supplementary Discussion

A core consideration of this work is the possibility of heating contributions. We begin by examining the possibility of heating on the microwave absorption signal. We perform field-swept microwave absorption spectroscopy of the NZAFO to determine if sample heating could explain the microwave power dependent lineshape of the microwave absorption response of the NZAFO. The microwave absorption measurements in this supplemental section were taken using magnetic field-modulation to enhance the signal-to-noise of the absorption signals. Thus, the uniform mode FMR response of the NZAFO appears as the derivative of a Lorentzian for low input microwave powers. A temperature change of the NZAFO sample would result in a change of the saturation magnetization and effective magnetic anisotropy fields, which would shift the FMR response to a different field. We investigate two possible ways that the NZAFO may undergo heating during measurement: resistive heating of the microstrip arising from the microwave currents in the microstrip antenna, and resonant absorptive microwave heating.

To investigate resistive heating of the NZAFO coming from the microstrip we measured the FMR response with microwaves on for a 100% duty cycle and compare with a 5% duty cycle at varying microwave powers. The result is shown in **Supplementary Figure 1**. For each input power we see that the FMR response of the NZAFO is identical, up to an overall multiplicative scaling which accounts for the microwave duty cycle. At low powers the FMR response is well characterized as a Lorentzian derivative centered on the uniform mode frequency. As microwave power is increased, the FMR line distorts toward lower magnetic field. If the shift toward lower magnetic field were caused by temperature changes related to microwave heating of the microstrip resonator, we would expect a different shift of the high microwave power FMR line for 5% duty cycle as compared to the 100% duty cycle response as the resistive heating of the microstrip would be considerably smaller for the low duty cycle. This suggests that the shift of the FMR signal toward lower field with increased microwave power is not coming from heating related to microwave currents in the microstrip.

Alternatively, the absorption of microwave energy by the spins may lead to an increase in the temperature of the NZAFO, which could alter the FMR response. This effect should be strongly dependent on the magnetic field sweep rate; a slow field sweep rate will allow more microwave energy to collect in the ferromagnet and will cause a greater shift in the NZAFO temperature, and thus would lead to a greater shift in the resonance condition as compared to a fast sweep. In **Supplementary Figure 1** we plot the FMR response of the NZAFO for high input microwave power for various magnetic field sweep rates. We find that there is no notable spectral change in the FMR signal at high microwave power as a function of sweep rate, thus, the shifted FMR line is not a result of absorptive microwave heating.

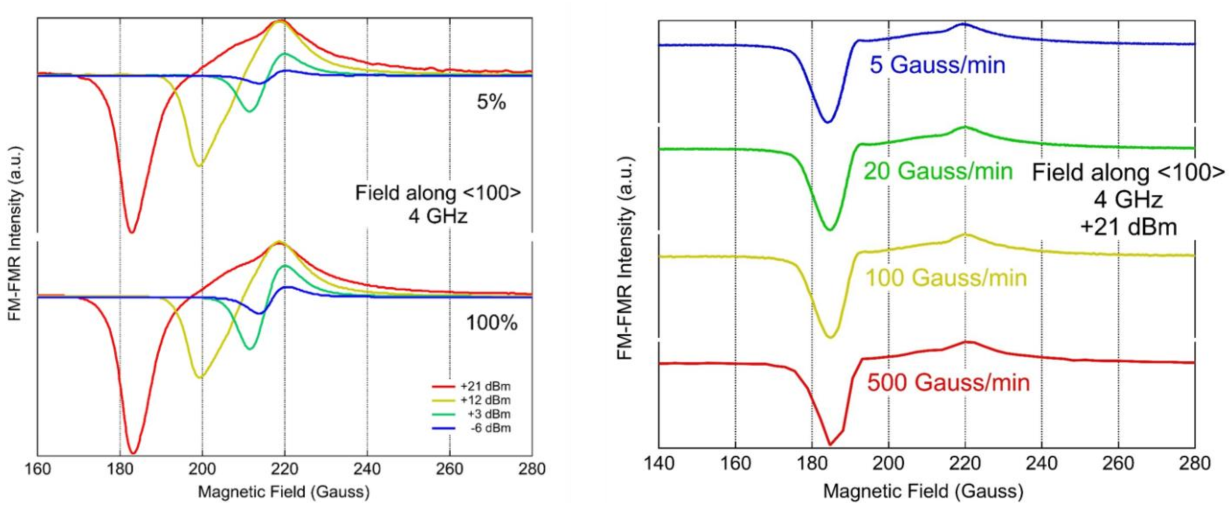

Supplementary Figure 1: Microwave Duty Cycle and Magnetic Field Sweep Rate Dependence

(left) Field-modulated FMR signal with the microwaves on for 5% (top) or 100% (bottom) of the total measurement time at varying input microwave powers. (right) FMR spectrum vs. magnetic field sweep rate in Gauss/min.

Next, we explore the possibility that an increase in NZAFO temperature could account for the magnetic field shift present in the broadband NV-detected FMR response. Having ruled out resistive and microwave absorption related heating, the only other mechanism for heating would be from the green laser used to address the NV spins.

In **Supplementary Figure 2** we show the high microwave power NV FMR response (+20 dBm, same data as **Figure 5(i)** of the main text) with the simultaneously-detected microwave absorption peaks marked with white circles. We performed field-modulated microwave absorption detected FMR of the NZAFO film at varying temperatures between 290 K and 350 K using a variable temperature broadband microwave absorption probe. These temperature dependent FMR data were taken at low microwave input power in order to best reflect the linear response of the NZAFO film as temperature is explicitly tuned. The temperature dependent microwave absorption data collected at 290 K matches well with the NZAFO microwave absorption signal which was taken simultaneously with the NV relaxometry. At low frequency (e.g. 2 GHz), the NZAFO FMR shifts toward low magnetic field with increased temperature. At high frequency (e.g. 4 GHz) the NZAFO FMR shifts toward higher magnetic field. These shifts are caused both by temperature dependence of the effective magnetization, and of the effective cubic anisotropy field. An increase in temperature decreases the cubic anisotropy which shifts the FMR line toward low field, but also decreases the magnetization, which will result in a shift the FMR line toward higher field. The competition of these two shifts results in the temperature dependence of the resonance condition at each temperature, magnetic field, and frequency.

We argue that the NV response is not related to heating of the NZAFO by considering the 4 GHz response of both the NV signal and the microwave absorption. We see from **Supplementary**

Figure 2 that an increase in NZAFO temperature leads to a shift of the FMR line to higher field. If the NZAFO were heated by the green laser during the collection of the NV data, we would expect the NV signal to reflect this elevated NZAFO temperature and trend on the high-field side of the FMR line at 4 GHz. This is exactly the opposite of what we see in **Supplementary Figure 2**. Additionally in **Figure 4** of the main text we see that the NV response to the driven NZAFO occurs at a magnetic field lower than the uniform mode response, at the second order spinwave instability shoulder.

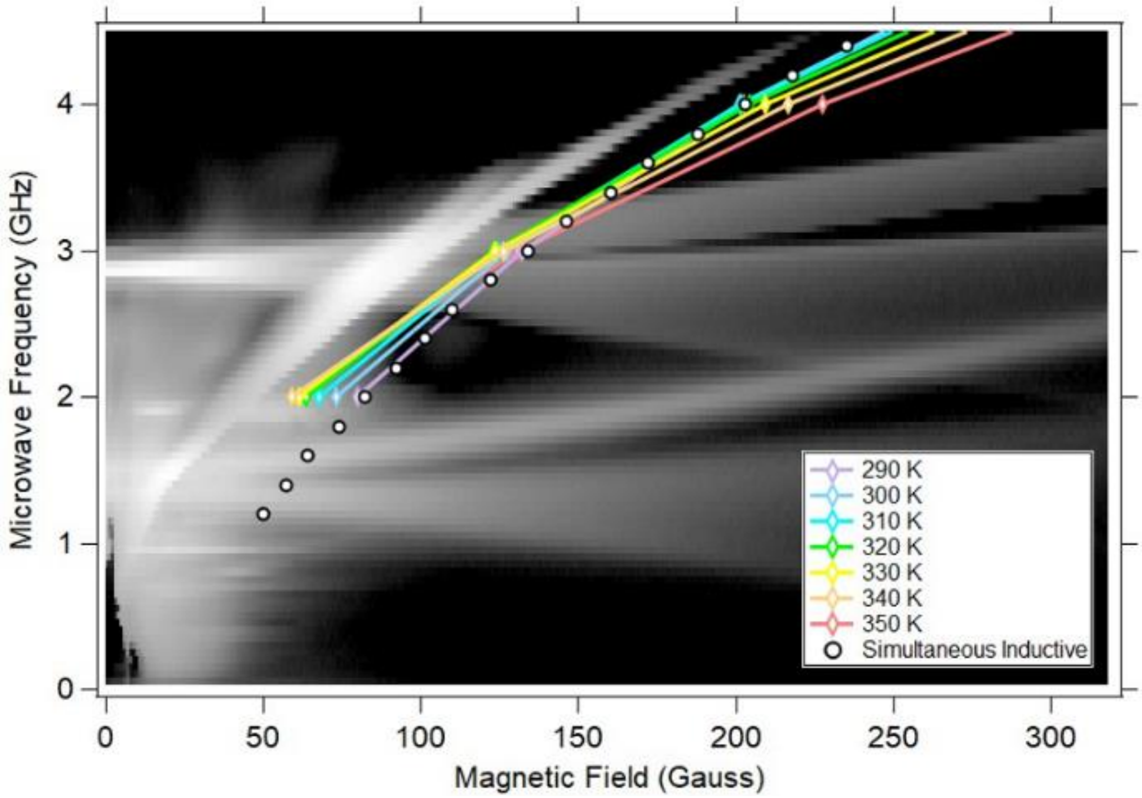

Supplementary Figure 2: Comparing high temperature NZAFO FMR with room temperature NV response

Temperature dependent FMR signal overlaid on room temperature NV signal (+20 dBm input microwave power). Temperature dependent FMR data at 290 K (purple diamonds) match the inductive FMR signal taken simultaneously with the NV signal (white circles).

Another central facet of this work is the calculation of the spinwave dispersion of NZAFO. Following Emori et al. [1] we can express the uniform mode ferromagnetic resonance condition of NZAFO as:

$$f(k=0) = \frac{\gamma}{2\pi}\sqrt{(H_{\mathrm{ext}} - H_{\mathrm{cub}})(H_{\mathrm{ext}} - H_{\mathrm{cub}} + H_{\mathrm{uni}} + 4\pi M_{\mathrm{s}})} \qquad (1)$$

where $\omega(k = 0)$ is the frequency of the uniform mode spinwave, k is the wavevector of the spinwave, $\frac{\gamma}{2\pi}$ = 3.2 MHz/Gauss (the g-factor of NZAFO has been shown to be ~2.29 in literature [1]), H_{ext} is the strength of the externally applied magnetic field in Gauss, H_{cub} is the strength of the effective cubic anisotropy field, H_{uni} is the strength of the effective uniaxial anisotropy field, $4\pi M_{\mathrm{s}}$ = 1500 Gauss is the literature value of the NZAFO saturation magnetization [1]. We determine the values of H_{cub} = 48.7 Gauss and H_{uni} = 8277 Gauss by fitting the uniform mode microwave absorption resonance condition at low input microwave power (e.g. **Figure 5(a)**) to **Equation (1)**.

Using the method of Kalinikos et al. [2] and including the relevant anisotropies [3] we can express the spinwave dispersion as

$$f(k) = \frac{\gamma}{2\pi} Sqrt\left[\left(H_{\mathrm{ext}} - H_{\mathrm{cub}} + Dk^2 + 4\pi M_{\mathrm{s}}(1-P)\right)\left(H_{\mathrm{ext}} - H_{\mathrm{cub}} + Dk^2 + 2\pi M_{\mathrm{s}} P\left(1 - Cos(2\phi_{\mathrm{k}})\right)\right)\right] \quad (2)$$

where $D = 3.4 \times 10^{-12}$ Gauss m^2 is the spinwave stiffness of NZAFO [4], ϕ_{k} is the angle of in-plane propagation of the spinwave relative to the magnetization direction, and $P = 1 - (1 - e^{-kd})/kd$ is an expression which accounts for the contribution of dipolar fields to the spinwave frequency. d = 23 nm is the NZAFO film thickness.

In **Figure 3(g)** and **Figure 4(e)** of the main text we plot **Equation (2)** for both the $\vec{\mathbf{k}} \parallel \vec{\mathbf{M}}$ and $\vec{\mathbf{k}} \perp \vec{\mathbf{M}}$ branches for some fixed value of the static applied magnetic field. The microwave drive frequency can be compared to these dispersions in order to determine the spinwave wavevector that is excited by the microwave drive, with the spinwaves being most easily excited by the second-order instability process for the $\vec{\mathbf{k}} \parallel \vec{\mathbf{M}}$ branch. We plot the intersection of the microwave drive frequency with this spinwave branch in **Figure 3(h)** and **Figure 4(f) of the main text.**

In **Figure 3(h)** we determine the range of spinwave wavevectors responsible for NV relaxation. To do so, we calculate the range of frequencies our NV powder ensemble will take for a given value of the applied field, using the NV Hamiltonian $\mathcal{H} = DS_{\mathrm{z}}^2 + \gamma_{\mathrm{NV}} \vec{B} \cdot \vec{S}$ where D is the ground state NV splitting of 2.87 GHz, γ_{NV} is the NV gyromagnetic ratio of 2.8 MHz/Gauss, and S_i are the spin-1 Pauli matrices. We calculate the upper and lower bounds of the NV frequency range by considering the frequency of NVs whose spins are collinear with the magnetic field. We then compare these upper and lower NV frequencies at each value of the magnetic field with the spinwave dispersion to quantify the range of spinwave wavevectors responsible for NV relaxation.

As mentioned in the main text, a microwave power correction was used for the broadband FMR and NV relaxometry signals. For the data in **Figure 5** of the main text we remove some of the frequency response of our microstrip antenna from the data. To do so, we first take a frequency sweep of the microstrip and recorded the microwave transmission amplitude at each frequency. We then determine which frequency between 50 MHz and 4.5 GHz corresponds to the minimum

microwave transmission amplitude for a fixed microwave power, and correspondingly lower the input microwave power at all other frequencies such that the transmission amplitude is approximately constant (within a few percent) across the entire frequency range of the measurement. The uncorrected microwave transmission for fixed microwave power and the corrected microwave transmission with the corresponding microwave input powers used to achieve constant microwave power transmission are shown in **Supplementary Figure 3**.

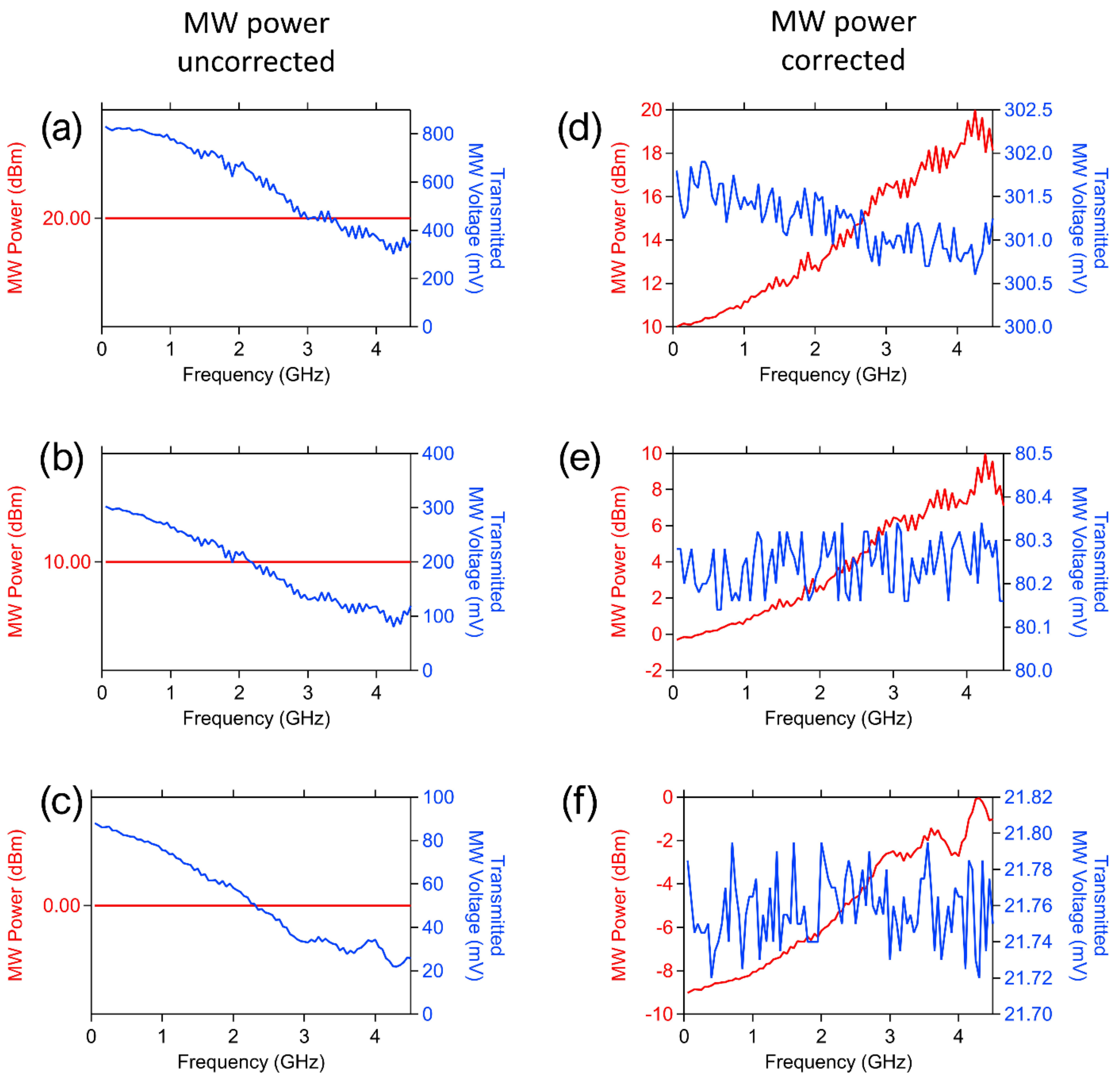

Supplementary Figure 3: Microwave power correction used for broadband FMR and NV measurements

Transmitted microwave power for fixed input microwave power versus microwave frequency at 20 dBm (a), 10 dBm (b), and 0 dBm (c). Corrected microwave transmission and corresponding microwave input power at "20 dbm" (d), "10 dbm" (e), and "0 dbm" (f).

Last, it is worthwhile to show the lockin PL response of nanodiamonds deposited on a non-magnetic insulating sample. Comparison of this data with the broadband NV data in the main text allows for clear understanding of which spectral features arise from the ferromagnet, and which are intrinsic to the NV spins.

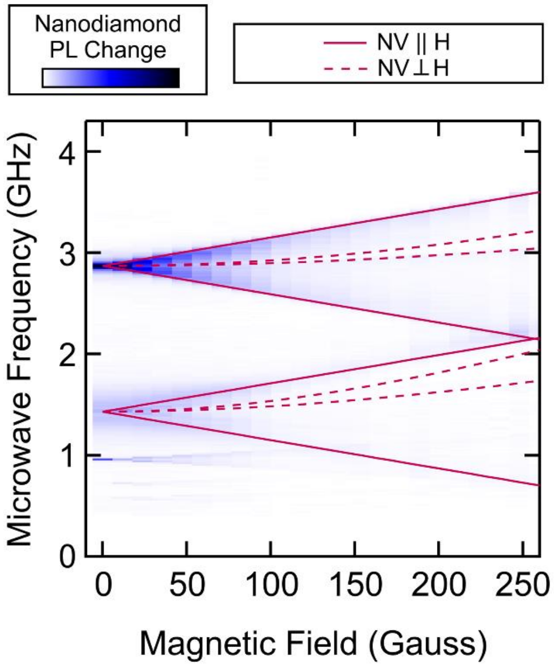

Supplementary Figure 4: NV nanodiamond ensemble PL response

Fluorescence contrast of a powder of NV nanodiamonds as a function of microwave frequency and magnetic field strength. Calculated ground and excited NV ESR frequencies overlaid.

Supplementary Figure 4 shows the PL response of an ensemble of randomly oriented NV nanodiamonds deposited on an insulating gallium gadolinium garnet substrate. Data are from the sample used in our previous manuscript.[5]

Supplementary References

1 Emori, S. et al. Coexistence of Low Damping and Strong Magnetoelastic Coupling in Epitaxial Spinel Ferrite Thin Films. *Advanced Materials* **29**, 1701130 (2017).

2 Kalinikos, B. A. & Slavin, A. N. Theory of dipole-exchange spin wave spectrum for ferromagnetic films with mixed exchange boundary conditions. *Journal of Physics C: Solid State Physics* **19**, 7013-7033 (1986).

3 Kalinikos, B. A., Kostylev, M. P., Kozhus, N. V. & Slavin, A. N. The dipole-exchange spin wave spectrum for anisotropic ferromagnetic films with mixed exchange boundary conditions. *Journal of Physics: Condensed Matter* **2**, 9861-9877 (1990).

4 Budhani, R. C. et al. Temperature dependent resonant microwave absorption in perpendicular magnetic anisotropy epitaxial films of a spinel ferrite. *Journal of Applied Physics* **125**, 243903 (2019).
5 Wolfe, C. S. et al. Off-resonant manipulation of spins in diamond via precessing magnetization of a proximal ferromagnet. *Physical Review B* **89** (2014).
